# The genome sequence of the orange ladybird, *Halyzia sedecimguttata* (Linnaeus, 1758)

**DOI:** 10.12688/wellcomeopenres.19369.1

**Published:** 2023-04-25

**Authors:** Liam M. Crowley, Maxwell Barclay, Helen E. Roy, Peter M.J. Brown, University of Oxford and Wytham Woods Genome Acquisition Lab, Natural History Museum Genome Acquisition Lab, Darwin Tree of Life Barcoding collective, Wellcome Sanger Institute Tree of Life programme, Wellcome Sanger Institute Scientific Operations: DNA Pipelines collective, Tree of Life Core Informatics collective, Darwin Tree of Life Consortium

**Affiliations:** 1 University of Oxford, Oxford, England, UK; 2 Natural History Museum, London, England, UK; 3 UK Centre for Ecology & Hydrology, Wallingford, England, UK; 4 School of Life Sciences, Anglia Ruskin University, Cambridge, England, UK

**Keywords:** Halyzia sedecimguttata, orange ladybird, genome sequence, chromosomal, Coleoptera

## Abstract

We present a genome assembly from an individual *Halyzia sedecimguttata *(the orange ladybird, Arthropoda; Insecta; Coleoptera, Coccinellidae). The genome sequence is 919.1 megabases in span. Most of the assembly is scaffolded into 10 chromosomal pseudomolecules, including the X sex chromosome. The mitochondrial genome has also been assembled and is 21.0 kilobases in length. Gene annotation of this assembly on Ensembl identified 27,547 protein coding genes.

## Species taxonomy

Eukaryota; Metazoa; Ecdysozoa; Arthropoda; Hexapoda; Insecta; Pterygota; Neoptera; Endopterygota; Coleoptera; Polyphaga; Cucujiformia; Coccinelloidea; Coccinellidae; Coccinellinae; Halyziini; *Halyzia*; *Halyzia sedecimguttata* (Linnaeus, 1758) (NCBI:txid347359).

## Background

The mildew-feeding orange ladybird, *Halyzia sedecimguttata*, was restricted to ancient woodland within the UK but in recent decades has been found on deciduous trees, including sycamore and ash, in many diverse habitats. Orange with sixteen white spots arranged in rows along the length, the adult beetle has distinct translucent edges. The larvae of the orange ladybird are almost fluorescent, with rows of black bristly tufts along the upper surface and black tips to the legs. The larvae are active much later than those of other ladybirds in the UK with the sculptured black pupae being seen into early winter.

Some people may have been fortunate to observe tiny yellow fruiting bodies on the surface of ladybirds. This stunning fungal parasite of ladybirds was classified as one species – *Hesperomyces virescens* Thaxt., but recently this fungus has been revealed, through multi-locus phylogenetic analyses coupled with sequence-based methods, to be a complex of many species ( Haelewaters *et al.*, 2018). One isolate collected from *Halyzia sedecimguttata* has been named *Hesperomyces halyziae* ( Haelewaters & Kesel, 2020) and is considered a sister to *H. virescens* s.l. from *Harmonia axyridis*, the harlequin ladybird. *Harmonia axyridis* is an invasive non-native species in many parts of the world ( Roy *et al.*, 2016) and appears to be causing distribution declines of *Halyzia sedecimguttata* and other ladybirds in the UK ( Roy *et al.*, 2012).

Several other ladybird genome assemblies have been published to date, including *Henosepilachna vigintioctomaculata* ( Zhu *et al.*, 2023), *Coccinella septempunctata* ( Crowley, 2021), *Propylea japonica* ( Zhang *et al.*, 2020), *Harmonia axyridis* ( Ando *et al.*, 2018; Boyes & Crowley, 2021; Chen *et al.*, 2021; Gautier *et al.*, 2018), *Adalia bipunctata* ( Goate, 2022) and *Cryptolaemus montrouzieri* ( Li *et al.*, 2021). Here we present a chromosomally complete genome sequence for *Halyzia sedecimguttata*, sequenced as part of the Darwin Tree of Life Project, which will also contribute to the growing set of genomic resources for Coccinellidae.

## Genome sequence report

**Figure 1.**
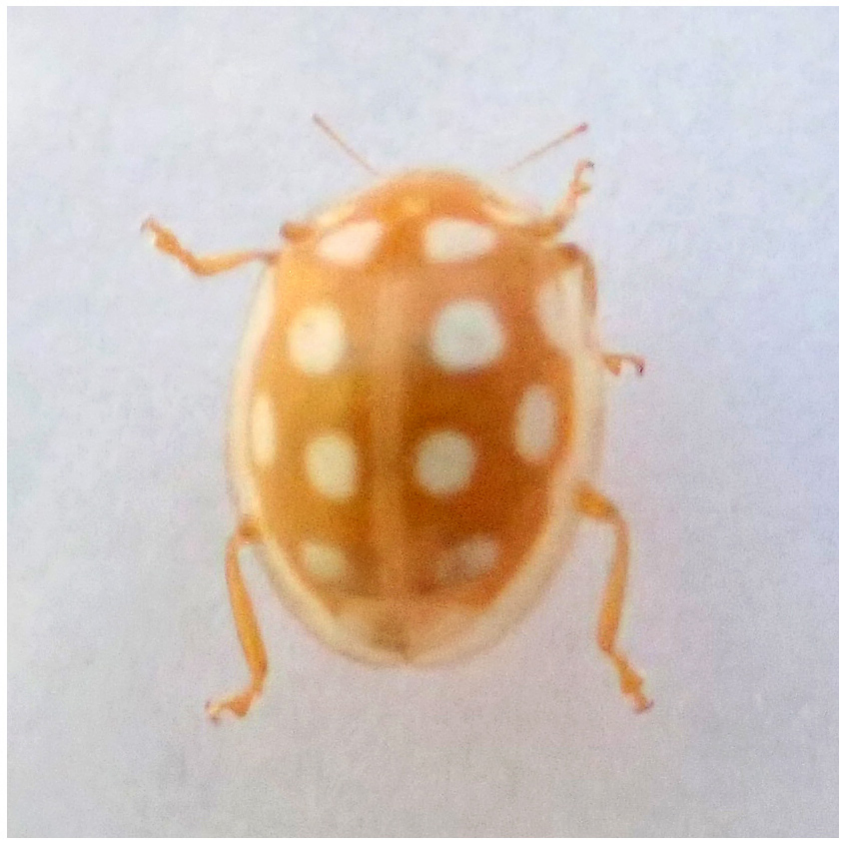
Photograph of the *Halyzia sedecimguttata* (icHalSede1) specimen used for genome sequencing.

The genome was sequenced from one *Halyzia sedecimguttata* specimen ( Figure 1) collected from Wytham Woods, Oxfordshire, UK (latitude 51.77, longitude –1.34). A total of 55-fold coverage in Pacific Biosciences single-molecule HiFi long reads and 46-fold coverage in 10X Genomics read clouds were generated. Primary assembly contigs were scaffolded with chromosome conformation Hi-C data. Manual assembly curation corrected 86 missing joins or mis-joins and removed 20 haplotypic duplications, reducing the assembly length by 1.38% and the scaffold number by 43.27%, and increasing the scaffold N50 by 0.16%]

**Figure 2.**
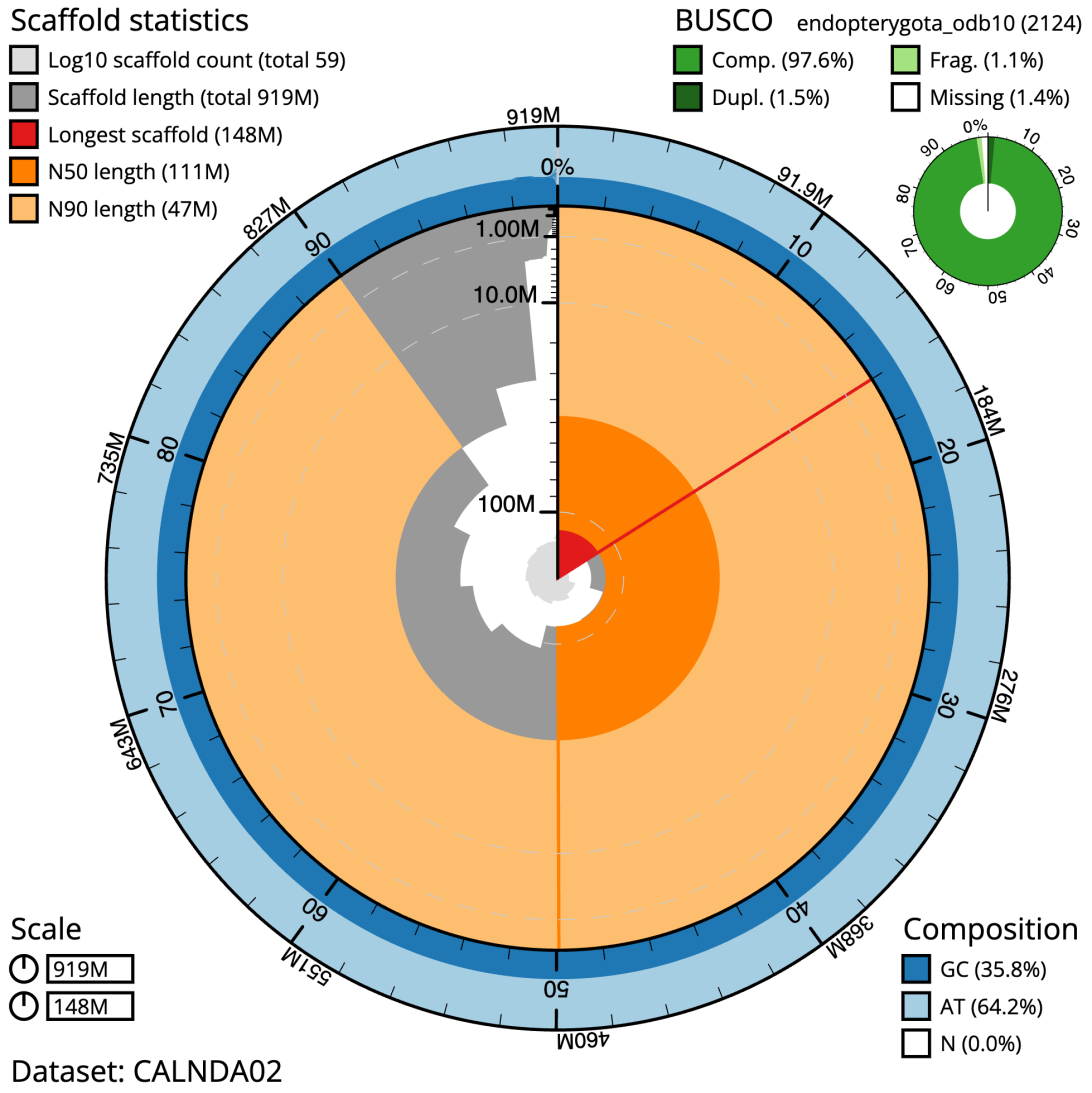
Genome assembly of *Halyzia sedecimguttata*, icHalSede1.2: metrics. The BlobToolKit Snailplot shows N50 metrics and BUSCO gene completeness. The main plot is divided into 1,000 size-ordered bins around the circumference with each bin representing 0.1% of the 919,111,547 bp assembly. The distribution of scaffold lengths is shown in dark grey with the plot radius scaled to the longest scaffold present in the assembly (147,779,010 bp, shown in red). Orange and pale-orange arcs show the N50 and N90 scaffold lengths (111,009,446 and 47,019,456 bp), respectively. The pale grey spiral shows the cumulative scaffold count on a log scale with white scale lines showing successive orders of magnitude. The blue and pale-blue area around the outside of the plot shows the distribution of GC, AT and N percentages in the same bins as the inner plot. A summary of complete, fragmented, duplicated and missing BUSCO genes in the endopterygota_odb10 set is shown in the top right. An interactive version of this figure is available at https://blobtoolkit.genomehubs.org/view/icHalSede1.2/dataset/CALNDA02/snail.

The final assembly has a total length of 919.1 Mb in 59 sequence scaffolds with a scaffold N50 of 111.0 Mb ( Table 1). Most (99.91%) of the assembly sequence was assigned to 10 chromosomal-level scaffolds, representing 9 autosomes and the X sex chromosome. Chromosome-scale scaffolds confirmed by the Hi-C data are named in order of size ( Figure 2– Figure 5; Table 2). Heterozygous inversions were observed at ~110 Mb on chromosome 2, ~57 Mb on chromosome 5 and ~26 Mb on chromosome 8. The specimen is most likely male, as there is half coverage of the X chromosome ( Figure 5), and karyotyping data indicate that the Y chromosome is very small.

**Table 1.** Genome data for *Halyzia sedecimguttata*, icHalSede1.2.

Project accession data		
Assembly identifier	icHalSede1.2	
Species	*Halyzia sedecimguttata*	
Specimen	icHalSede1	
NCBI taxonomy ID	347359	
BioProject	PRJEB51270	
BioSample ID	SAMEA8563708	
Isolate information	icHalSede1 (genome sequencing, Hi-C) idHalSede3 (RNA sequencing)	
Assembly metrics *		*Benchmark*
Consensus quality (QV)	64.3	*≥ 50*
*k*-mer completeness	100%	*≥ 95%*
BUSCO **	C:97.6%[S:96.1%,D:1.5%], F:1.1%,M:1.4%,n:2124	*C ≥ 95%*
Percentage of assembly mapped to chromosomes	99.91%	*≥ 95%*
Sex chromosomes	X chromosome	*localised homologous pairs*
Organelles	Mitochondrial genome assembled	*complete single alleles*
Raw data accessions		
PacificBiosciences SEQUEL II	ERR9127943, ERR9127944, ERR9468772	
10X Genomics Illumina	ERR9123835–ERR9123838	
Hi-C Illumina	ERR9123834	
PolyA RNA-Seq Illumina	ERR10123687	
**Genome assembly**		
Assembly accession	GCA_937662695.2	
*Accession of alternate haplotype*	GCA_937616445.2	
Span (Mb)	919.1	
Number of contigs	214	
Contig N50 length (Mb)	14.0	
Number of scaffolds	59	
Scaffold N50 length (Mb)	111.0	
Longest scaffold (Mb)	147.8	
**Genome annotation**		
Number of protein-coding genes	27,542	
Number of gene transcripts	27,825	

* Assembly metric benchmarks are adapted from column VGP-2020 of “Table 1: Proposed standards and metrics for defining genome assembly quality” from ( Rhie *et al.*, 2021). ** BUSCO scores based on the endopterygota_odb10 BUSCO set using v5.3.2. C = complete [S = single copy, D = duplicated], F = fragmented, M = missing, n = number of orthologues in comparison. A full set of BUSCO scores is available at https://blobtoolkit.genomehubs.org/view/icHalSede1.2/dataset/CALNDA02/busco.

**Figure 3.**
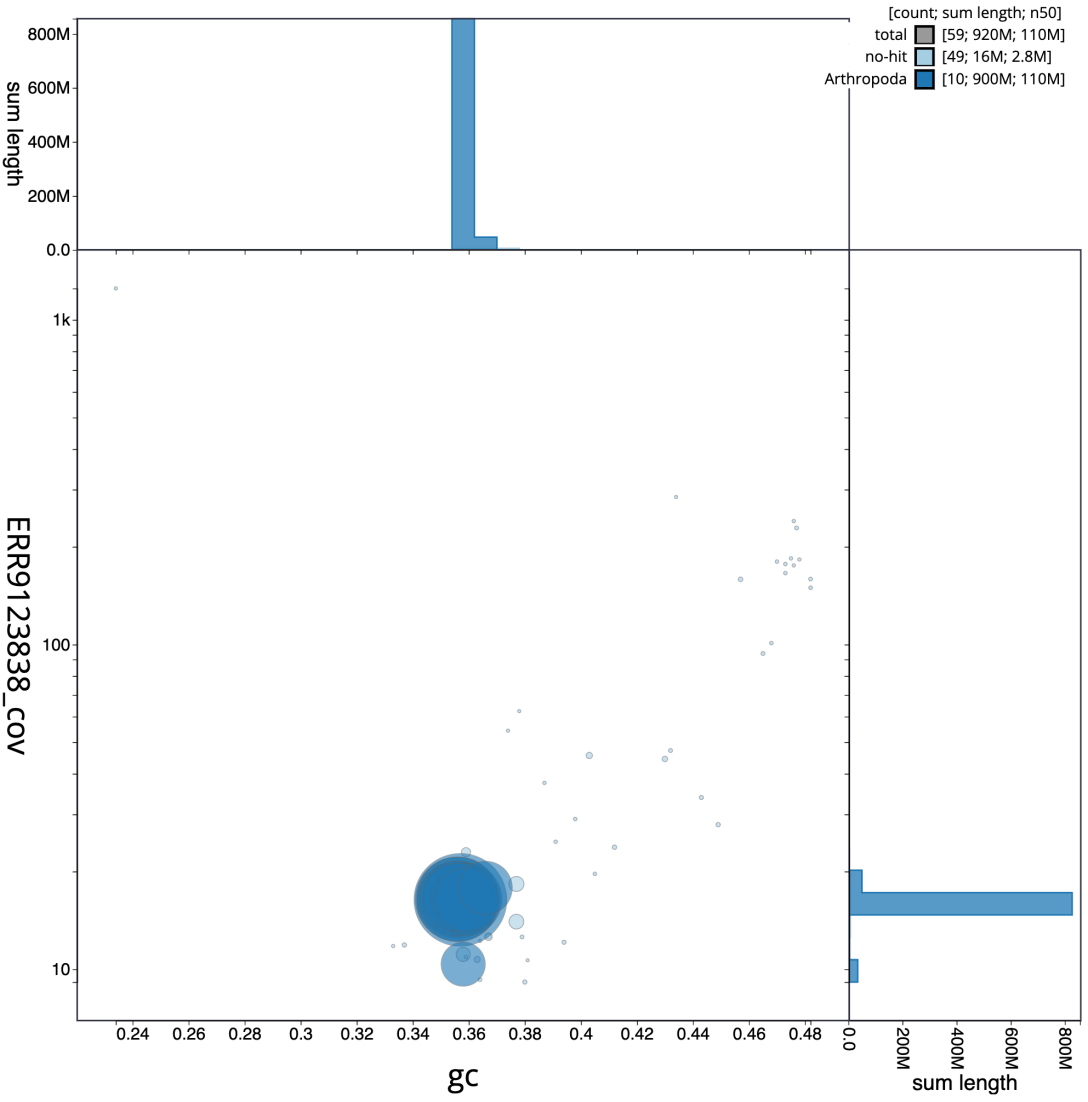
Genome assembly of *Halyzia sedecimguttata*, icHalSede1.2: BlobToolKit GC-coverage plot. Scaffolds are coloured by phylum. Circles are sized in proportion to scaffold length. Histograms show the distribution of scaffold length sum along each axis. An interactive version of this figure is available at https://blobtoolkit.genomehubs.org/view/icHalSede1.2/dataset/CALNDA02/blob.

**Figure 4.**
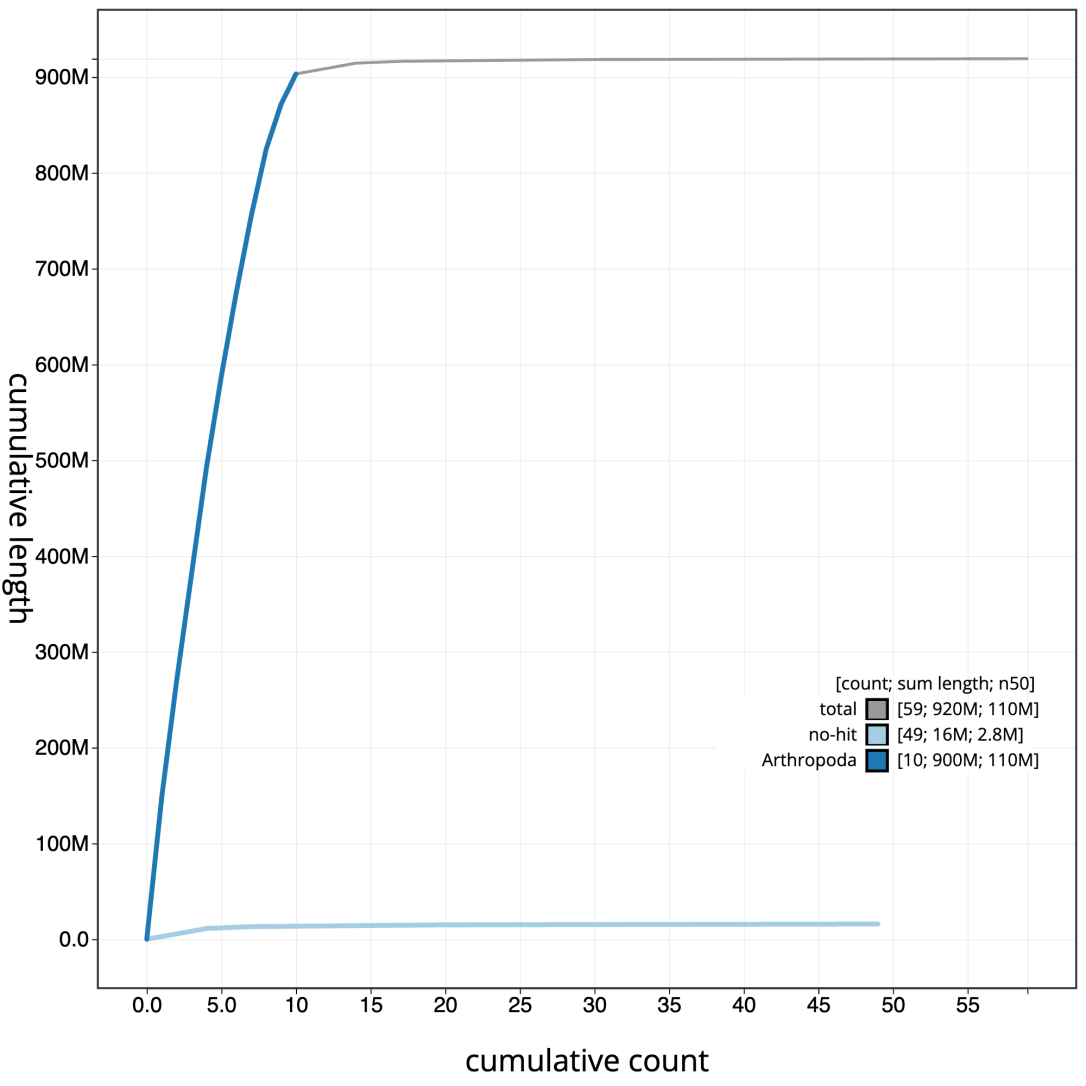
Genome assembly of *Halyzia sedecimguttata*, icHalSede1.2: BlobToolKit cumulative sequence plot. The grey line shows cumulative length for all scaffolds. Coloured lines show cumulative lengths of scaffolds assigned to each phylum using the buscogenes taxrule. An interactive version of this figure is available at https://blobtoolkit.genomehubs.org/view/icHalSede1.2/dataset/CALNDA02/cumulative.

**Figure 5.**
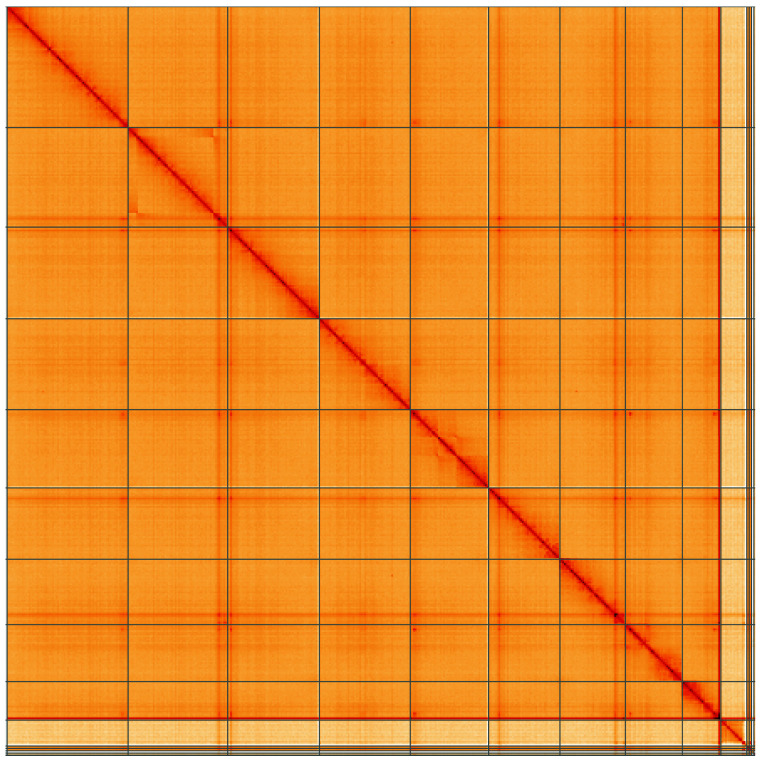
Genome assembly of *Halyzia sedecimguttata*, icHalSede1.2: Hi-C contact map of the icHalSede1.2 assembly, visualised using HiGlass. Chromosomes are shown in order of size from left to right and top to bottom. An interactive version of this figure may be viewed at https://genome-note-higlass.tol.sanger.ac.uk/l/?d=S4GocVVeSxuEIeCjWZ7r3w.

**Table 2.** Chromosomal pseudomolecules in the genome assembly of *Halyzia sedecimguttata*, icHalSede1.

INSDC accession	Chromosome	Size (Mb)	GC (%)
OW569411.2	1	147.78	35.5
OW569412.2	2	121.60	35.5
OW569413.2	3	111.96	35.5
OW569414.2	4	111.01	35.5
OW569415.2	5	95.57	35.5
OW569416.2	6	87.19	35.5
OW569417.2	7	79.88	35.5
OW569418.2	8	69.57	35.5
OW569419.2	9	47.02	36.5
OW569420.2	X	31.66	35.5
OW569421.2	MT	0.02	23

While not fully phased, the assembly deposited is of one haplotype. Contigs corresponding to the second haplotype have also been deposited. The mitochondrial genome was also assembled and can be found as a contig within the multifasta file of the genome submission.

The estimated Quality Value (QV) of the final assembly is 64.3 with *k*-mer completeness of 100%, and the assembly has a BUSCO v5.3.2 completeness of 97.6% (single = 96.1%, duplicated = 1.5%), using the endopterygota_odb10 reference set ( *n* = 2,124).

Metadata for specimens, spectral estimates, sequencing runs, contaminants and pre-curation assembly statistics can be found at https://links.tol.sanger.ac.uk/species/347359.

## Genome annotation report

The *Halyzia sedecimguttata* GCA_937662695.1 genome assembly was annotated using the Ensembl rapid annotation pipeline ( Table 1; https://rapid.ensembl.org/Halyzia_sedecimguttata_GCA_937662695.1/Info/Index). The resulting annotation includes 27,825 transcribed mRNAs from 27,547 protein-coding genes.

## Methods

### Sample acquisition and nucleic acid extraction

A *Halyzia sedecimguttata* specimen (icHalSede1) was collected from Wytham Woods, Oxfordshire (biological vice-county: Berkshire), UK (latitude 51.77, longitude –1.34) on 29 January 2020. The specimen was found on a beech trunk by Liam Crowley (University of Oxford) and collected by potting. The specimen was identified by the collector and then snap-frozen on dry ice. This specimen was used for genome sequencing and Hi-C scaffolding.

A second *Halyzia sedecimguttata* specimen (icHalSede3) was collected from Parsons Green, London UK (latitude 51.48, longitude –0.18). The specimen was potted by Maxwell Barclay (Natural History Museum). The specimen was identified by the collector and then snap-frozen at –80°C. This specimen was used for RNA sequencing.

DNA was extracted at the Tree of Life laboratory, Wellcome Sanger Institute (WSI). The icHalSede1 sample was weighed and dissected on dry ice, setting aside tissue for Hi-C sequencing. Whole organism tissue was disrupted using a Nippi Powermasher fitted with a BioMasher pestle. High molecular weight (HMW) DNA was extracted using the Qiagen MagAttract HMW DNA extraction kit. Low molecular weight DNA was removed from a 20 ng aliquot of extracted DNA using the 0.8X AMpure XP purification kit prior to 10X Chromium sequencing; a minimum of 50 ng DNA was submitted for 10X sequencing. HMW DNA was sheared into an average fragment size of 12–20 kb in a Megaruptor 3 system with speed setting 30. Sheared DNA was purified by solid-phase reversible immobilisation using AMPure PB beads with a 1.8X ratio of beads to sample to remove the shorter fragments and concentrate the DNA sample. The concentration of the sheared and purified DNA was assessed using a Nanodrop spectrophotometer and Qubit Fluorometer and Qubit dsDNA High Sensitivity Assay kit. Fragment size distribution was evaluated by running the sample on the FemtoPulse system.

RNA was extracted from abdomen tissue of icHalSede3 in the Tree of Life Laboratory at the WSI using TRIzol, according to the manufacturer’s instructions. RNA was eluted in 50 μl RNAse-free water and its concentration assessed using a Nanodrop spectrophotometer and Qubit Fluorometer using the Qubit RNA Broad-Range (BR) Assay kit. Analysis of the integrity of the RNA was done using Agilent RNA 6000 Pico Kit and Eukaryotic Total RNA assay.

### Sequencing

Pacific Biosciences HiFi circular consensus and 10X Genomics read cloud DNA sequencing libraries were constructed according to the manufacturers’ instructions. Poly(A) RNA-Seq libraries were constructed using the NEB Ultra II RNA Library Prep kit. DNA and RNA sequencing were performed by the Scientific Operations core at the WSI on Pacific Biosciences SEQUEL II (HiFi) and Illumina NovaSeq 6000 (RNA-Seq and 10X) instruments. Hi-C data were also generated from tissue of icHalSede3 that had been set aside, using the Arima2 kit and sequenced on the Illumina NovaSeq 6000 instrument.

### Genome assembly, curation and evaluation

Assembly was carried out with Hifiasm ( Cheng *et al.*, 2021) and haplotypic duplication was identified and removed with purge_dups ( Guan *et al.*, 2020). One round of polishing was performed by aligning 10X Genomics read data to the assembly with Long Ranger ALIGN, calling variants with FreeBayes ( Garrison & Marth, 2012). The assembly was then scaffolded with Hi-C data ( Rao *et al.*, 2014) using YaHS ( Zhou *et al.*, 2023). The assembly was checked for contamination as described previously ( Howe *et al.*, 2021). Manual curation was performed using HiGlass ( Kerpedjiev *et al.*, 2018) and Pretext ( Harry, 2022). The mitochondrial genome was assembled using MitoHiFi ( Uliano-Silva *et al.*, 2022), which runs MitoFinder ( Allio *et al.*, 2020) or MITOS ( Bernt *et al.*, 2013) and uses these annotations to select the final mitochondrial contig and to ensure the general quality of the sequence. To evaluate the assembly, MerquryFK was used to estimate consensus quality (QV) scores and *k*-mer completeness ( Rhie *et al.*, 2020). The genome was analysed within the BlobToolKit environment ( Challis *et al.*, 2020) and BUSCO scores ( Manni *et al.*, 2021; Simão *et al.*, 2015) were calculated. Table 3 contains a list of software tool versions and sources.

**Table 3.** Software tools: versions and sources.

Software tool	Version	Source
BlobToolKit	4.1.2	https://github.com/blobtoolkit/blobtoolkit
BUSCO	5.3.2	https://gitlab.com/ezlab/busco
FreeBayes	1.3.1-17-gaa2ace8	https://github.com/freebayes/freebayes
Hifiasm	0.16.1-r375	https://github.com/chhylp123/hifiasm
HiGlass	1.11.6	https://github.com/higlass/higlass
Long Ranger ALIGN	2.2.2	https://support.10xgenomics.com/genome-exome/software/pipelines/latest/advanced/other-pipelines
Merqury	MerquryFK	https://github.com/thegenemyers/MERQURY.FK
MitoHiFi	2	https://github.com/marcelauliano/MitoHiFi
PretextView	0.2	https://github.com/wtsi-hpag/PretextView
purge_dups	1.2.3	https://github.com/dfguan/purge_dups
YaHS	yahs-1.1.91eebc2	https://github.com/c-zhou/yahs

### Genome annotation

The BRAKER2 pipeline ( Brůna *et al.*, 2021) was used in the default protein mode to generate annotation for the *Halyzia sedecimguttata* assembly (GCA_937662695.1). in Ensembl Rapid Release.

### Ethics and compliance issues

The materials that have contributed to this genome note have been supplied by a Darwin Tree of Life Partner. The submission of materials by a Darwin Tree of Life Partner is subject to the Darwin Tree of Life Project Sampling Code of Practice. By agreeing with and signing up to the Sampling Code of Practice, the Darwin Tree of Life Partner agrees they will meet the legal and ethical requirements and standards set out within this document in respect of all samples acquired for, and supplied to, the Darwin Tree of Life Project. All efforts are undertaken to minimise the suffering of animals used for sequencing. Each transfer of samples is further undertaken according to a Research Collaboration Agreement or Material Transfer Agreement entered into by the Darwin Tree of Life Partner, Genome Research Limited (operating as the Wellcome Sanger Institute), and in some circumstances other Darwin Tree of Life collaborators.

## Data Availability

European Nucleotide Archive: *Halyzia sedecimguttata* (orange ladybird). Accession number PRJEB51270; https://identifiers.org/ena.embl/PRJEB51270. ( Wellcome Sanger Institute, 2022) The genome sequence is released openly for reuse. The *Halyzia sedecimguttata* genome sequencing initiative is part of the Darwin Tree of Life (DToL) project. All raw sequence data and the assembly have been deposited in INSDC databases. Raw data and assembly accession identifiers are reported in Table 1.

## Author information

Members of the University of Oxford and Wytham Woods Genome Acquisition Lab are listed here: https://doi.org/10.5281/zenodo.4789928.

Members of the Natural History Museum Genome Acquisition Lab are listed here: https://doi.org/10.5281/zenodo.4790043.

Members of the Darwin Tree of Life Barcoding collective are listed here: https://doi.org/10.5281/zenodo.4893703.

Members of the Wellcome Sanger Institute Tree of Life programme are listed here: https://doi.org/10.5281/zenodo.4783585.

Members of Wellcome Sanger Institute Scientific Operations: DNA Pipelines collective are listed here: https://doi.org/10.5281/zenodo.4790455.

Members of the Tree of Life Core Informatics collective are listed here: https://doi.org/10.5281/zenodo.5013541.

Members of the Darwin Tree of Life Consortium are listed here: https://doi.org/10.5281/zenodo.4783558.
