# Supplementary material for: The genome sequence of the orange ladybird, Halyzia sedecimguttata (Linnaeus, 1758)
Source: Wellcome Open Res. 2023 Apr 25;8:186. [Version 1] doi: 10.12688/wellcomeopenres.19369.1 (PMC10354457; doi:10.12688/wellcomeopenres.19369.1)
Supplement: Peer review history

# Peer review: Reviewer response for version 1

**DOI:** 10.21956/wellcomeopenres.21456.r62397

**Published:** 2023-07-18

**Authors:** Camille Meslin

**Affiliations:** 1 Institute of Ecology and Environmental Sciences of Paris, iEES-Paris, INRAE, Sorbonne Université, CNRS, IRD, UPEC, Université Paris Diderot, 75013 Versailles, France

The authors are presenting a very clear data note on the genome sequence of the orange ladybug *Halyzia sedecimguttata*. The genome sequence is of impressive quality with a chromosome-level assembly. For that the authors used state-of-the-art methodologies (combining long reads and physical mapping).

 Two minor points need clarification:

1. Which tissue was set aside from specimen #3 and used later for HiC?
2. A tool was used to identify contaminations but there is no report on that in the manuscript, does this mean that there was no contamination at all?

Are sufficient details of methods and materials provided to allow replication by others?

Yes

Is the rationale for creating the dataset(s) clearly described?

Yes

Are the datasets clearly presented in a useable and accessible format?

Yes

Are the protocols appropriate and is the work technically sound?

Yes

Reviewer Expertise:

Insect chemosensory receptor evolution

I confirm that I have read this submission and believe that I have an appropriate level of expertise to confirm that it is of an acceptable scientific standard.

# Peer review: Reviewer response for version 1

**DOI:** 10.21956/wellcomeopenres.21456.r57089

**Published:** 2023-05-30

**Authors:** Zachary Cohen

**Affiliations:** 1 Insect Control and Cotton Disease Research Unit, Southern Plains Agricultural Research Center,Agricultural Research Service, United States Department of Agriculture, College Station, TX, USA

This was a well-executed and thorough genome report. I commend the authors on their efforts and utility of the most cutting-edge resources but would have liked to see more scrutiny and elaboration of the annotated/transcribed genes and their putative role in: 1. mildew feeding 2. immunity/fungal pathogen defense and 3. invasiveness. Elaborating on these traits and features unique to this system, I feel, is sufficient but not necessary for this to be indexed.

Are sufficient details of methods and materials provided to allow replication by others?

Yes

Is the rationale for creating the dataset(s) clearly described?

Yes

Are the datasets clearly presented in a useable and accessible format?

Yes

Are the protocols appropriate and is the work technically sound?

Yes

Reviewer Expertise:

Entomology, genomics, comparative genetics, bioinformatics, phylogenetics, evolution.

I confirm that I have read this submission and believe that I have an appropriate level of expertise to confirm that it is of an acceptable scientific standard.
